# *Moringa oleifera* Hot Water Extract Protects Vero Cells from Hydrogen Peroxide-Induced Oxidative Stress by Regulating Mitochondria-Mediated Apoptotic Pathway and Nrf2/HO-1 Signaling

**DOI:** 10.3390/foods11030420

**Published:** 2022-01-31

**Authors:** Kirinde Gedara Isuru Sandanuwan Kirindage, Ilekuttige Priyan Shanura Fernando, Arachchige Maheshika Kumari Jayasinghe, Eui-Jeong Han, Mawalle Kankanamge Hasitha Madhawa Dias, Kyung-Pil Kang, Sung-Ig Moon, Tai-Sun Shin, Ayeong Ma, Ginnae Ahn

**Affiliations:** 1 Department of Food Technology and Nutrition, Chonnam National University, Yeosu 59626, Korea; 218388@jnu.ac.kr (K.G.I.S.K.); 218385@jnu.ac.kr (A.M.K.J.); iosu5772@naver.com (E.-J.H.); 198807@jnu.ac.kr (M.K.H.M.D.); 2 Department of Marine Bio-Food Sciences, Chonnam National University, Yeosu 59626, Korea; shanurabru@gmail.com; 3 Research Center for Healthcare and Biomedical Engineering, Chonnam National University, Yeosu 59626, Korea; 4 Jeju Changhae Fisheries Co., Ltd., Jeju 63072, Korea; golf87@naver.com (K.-P.K.); jejuchanghae@naver.com (S.-I.M.); 5 Department of Food Science and Nutrition, Chonnam National University, 77 Yongbong-ro, Buk-gu, Gwangju 61186, Korea; shints@chonnam.ac.kr (T.-S.S.); ayeong_ma@daum.net (A.M.)

**Keywords:** *Moringa oleifera*, oxidative stress, antioxidants, Vero, polyphenols

## Abstract

The present study discloses the identification of phenolic compounds in *Moringa oleifera* hot water extract (MOH) and the evaluation of its antioxidant activity on H_2_O_2_-induced oxidative stress in Vero cells. Upon analysis, MOH was found to contain phenolic compounds and indicated 2,2-diphenyl-1-picrylhydrazyl (DPPH) and 2,2-azino-bis (3-ethylbenzothiazoline-6-sulfonic acid) diammonium salt (ABTS^+^) radical scavenging with IC_50_ values of 102.52 and 122.55 µg/mL, respectively. The ferric reducing antioxidant power (FRAP) of MOH indicated a dose-dependent increase with a maximum absorbance at 125 μg/mL and the oxygen radical absorbance capacity (ORAC) of MOH was 1004.95 µmol TE/mg. Results showed that MOH dose-dependently reduced intracellular ROS generation in H_2_O_2_-stimulated Vero cells while increasing the cell viability. Fluorescence microscopy and flowcytometric analyses have supported the above findings. MOH markedly suppressed the H_2_O_2_-induced mitochondrial depolarization and apoptosis through suppression of the mitochondrial-mediated apoptosis pathway and activated the Nrf2/HO-1 signaling pathway by possibly involving H_2_O_2_ generation in cell media. Findings of western blot were supported by immunocytochemistry of Nrf2 nuclear translocation. Thus, MOH bioactivity would potentiate its applications in manufacturing functional food.

## 1. Introduction

Exposure to ionizing radiation, xenobiotics, and disease conditions generate oxidative stress in live cells, which has been linked to the pathogenesis of a variety of detrimental cellular responses [1]. As evidence accumulated over time has shown, oxidative and inflammatory processes in the human body are triggered by lifestyle-related factors such as exposure to contaminated air, smoke and alcohol consumption, exposure to ionization radiation, xenobiotics, and urbanization hypoxia [2]. Cell and tissue damage, and, hence, the development of non-communicable diseases, are primarily caused by oxidative stress and chronic inflammatory processes [2]. The dysregulated production of reactive oxygen species (ROS) in cells is a well-known reason for oxidative stress and oxidative stress in cells is exacerbated when oxidation reactions are prevalent in the organism [3].

The cellular enzymatic and non-enzymatic oxidant and antioxidant systems play a pivotal role in neutralizing superoxide anion radicals, hydrogen peroxide, and hydroxyl radicals, as well as secondary reactive species such as peroxyl and alkoxyl radicals generated by subsequent oxidation in live cells. Superoxide dismutase converts anion radicals, which are generated by a series of enzymes, into intracellular hydrogen peroxide in the cell environment. Moreover, hydrogen peroxide in cells can be produced by enzymes such as nicotine adenine dinucleotide phosphate oxidase, xanthine oxidase, and amino acid oxidase, as well as increased oxygen consumption in metabolic processes in the peroxisome [4]. Even so, cells are equipped with an antioxidant defense system such as peroxiredoxins and glutathione peroxidases, which catalyzes the removal of H_2_O_2_ to maintain their levels at physiological concentration [5]. If this is unable to convert hydrogen peroxide to water and oxygen, then hydrogen peroxide can react with superoxide radicals or undergo Fenton reactions or Haber–Weiss reactions to generate hydroxyl radicals, which can wreak havoc on cells by causing oxidative stress [4]. Apart from that, the mitochondrial electron transport system acts as a primary endogenous ROS generation site in live cells. Moreover, chemicals such as H_2_O_2_ may readily diffuse across the cell membrane, react with intracellular ions, and cause intracellular damage [6]. ROS is linked to a variety of complications, including cancer, heart disease, neurological illnesses, and infertility, due to its potential to damage nucleic acid, protein alterations, and lipid peroxidation [7,8]. When it comes to the liver, a disturbed balance of pro and antioxidants has been found as a risk factor for liver cancer progression [9]. Moreover, ROS causes the pathogenesis of acute kidney injury and its transition to chronic kidney disease [10,11,12]. Hence, research into naturally occurring antioxidants for the prevention of oxidative stress and related health disorders has received a lot of attention in recent years [13]. The strong antioxidants with low toxicity are potentially beneficial for humans as supplementary antioxidants for remediating the effect of accumulated ROS in cells [6]. Aside from that, natural alternatives to synthetic flavor enhancers and antioxidants in the food business are becoming increasingly popular to prevent oxidation and preserve sensory qualities [14].

A wide range of antioxidants in food and medicinal plants have been identified over the decades and they can be split into two groups based on their polarity as ‘water-soluble’ and ‘fat-soluble’ antioxidants. Phenols, flavonoids, anthocyanins, stilbenes, and ligands are water-soluble antioxidants, while α-carotene, β-carotene, lycopene, lutein, and zeaxanthin are lipo-soluble antioxidants found primarily in plants [15]. The dietary phytonutrients, including *Moringa oleifera* flavonoids, were of major interest in the study because of their nutritional properties, potential anti-inflammatory and antioxidant properties, and their aptitude to prevent normal cell DNA damage and to encourage cancer cell death as a therapeutic input [16].

*M. oleifera*, generally known as ‘Moringa’, is one of the well-known sources of biocompatible antioxidants that abundantly grow in semiarid, tropical, and subtropical areas [17]. Cooked drumsticks are known to be popular in many countries including South Asia and some African countries; however, leaves are not popular as a food commodity widely. As well as this, *M. oleifera* is a prevalent ingredient in traditional medicine in many countries, where it has promised effects on various chronic diseases [18]. Numerous research have confirmed the presence of phenolic compounds such as anthocyanins and flavonoids in *M. oleifera* leaves, which are responsible for its strong antioxidant free radical scavenging and antidiabetic activities [17,19]. According to the present understanding, *M. oleifera* can be used as a treatment for a variety of conditions related to heart disease, diabetes, cancer, and fatty liver [20]. The effect of *M. oleifera* hot water extract on major organs has previously been investigated using rat models to measure lipid peroxide levels [21]. However, certain gaps in understanding may be filled by taking on further studies on Moringa.

As oxidative stress has implications for human kidneys, Vero cells derived from African green monkey kidney fibroblasts were used in assessing oxidative stress using H_2_O_2_ to generate ROS in cells [22]. The present study was carried out to evaluate the efficacy of the hot water extraction method to extract *M. oleifera* and evaluate the antioxidant activity of *M. oleifera* hot water extract (MOH) on H_2_O_2_-induced oxidative stress in Vero cells. The study was carried out by hypothesizing that MOH ameliorates the effects of H_2_O_2_-induced oxidative stress in Vero cells by suppressing the mitochondria-mediated apoptosis pathway and promoting the Nrf2/HO-1 signaling pathway.

## 2. Materials and Methods

### 2.1. Materials

*M. oleifera* was collected from Suncheon Bay Moringa Cooperative (Suncheon-si, Jeollanam-do, Korea). Dulbecco’s modified eagle medium (DMEM), and a mixture of streptomycin and penicillin (P/S) as antibiotics were purchased from GibcoBRL (Grand Island, NY, USA). Fetal bovine serum (FBS) was purchased from Welgene (Gyeongsangbuk-do, South Korea). 2,2-azino-bis(3-ethylbenzothiazoline-6-sulfonic acid) diammonium salt (ABTS), 2,2-diphenyl-1-picrylhydrazyl (DPPH), 2′7′-dichlorodihydrofluorescein diacetate (DCFH-DA), 3-(4,5-dimethylthiazol-2-yl)-2,5-diphenyltetrazolium bromide (MTT), Dimethyl sulfoxide (DMSO), bovine serum albumin (BSA), Folin and Ciocalteu’s phenol reagent, ethidium bromide, agarose, 2,2′-azobis(2-amidino-propane) dihydrochloride (AAPH), fluorescein sodium, 6-Hydroxy-2,5,7,8-tetramethylchroman-2-carboxylic acid (Trolox), gallic acid, D-mandelic acid, 2,3,4-trihydroxy benzoic acid, 3,4-dihydroxy benzaldehyde, 4-hydroxy benzoic acid, gentisic acid sodium salt hydrate, catechin hydrate, vanillic acid, 3-hydroxy benzoic acid, chlorogenic acid, syringic acid, p-coumaric acid, 3,4-dimethoxy benzoic acid, sinapic acid, rutin hydrate, trans-cinnamic acid, and quercetin were bought from Sigma-Aldrich (St. Louis, MO, USA). D-glucose was purchased from Junsei Chemical Co., Ltd. (Tokyo, Japan). JC-1 Assay kit was obtained from Thermo Fisher Scientific (Waltham, MA, USA) and Alexa Fluor^®^ 488 conjugated Anti-Mouse IgG secondary antibody was purchased from Cell Signaling Technologies (Bedford, MA, USA). Protein assay kit, NE-PER^®^ nuclear and cytoplasmic extraction kit, 1-Step transfer buffer, Pierce™ RIPA buffer, protein ladder, and SuperSignal™ West Femto Maximum Sensitivity Substrate were purchased from Thermo Fisher Scientific (Rockford, IL, USA). Antibodies needed for the western blot analysis were purchased from Santa Cruz Biotechnology Inc. (Dallas, TX, USA) and Cell Signaling Technology Inc. (Beverly, MA, USA). Skim milk powder was obtained from BD Difco™ (Sparks, MD, USA). Normal goat serum, Prolong^®^ Gold antifade reagent with DAPI reagent, and DyLihgtTM 554 Phalloidin were purchased from Cell Signaling Technology (Danvers, MA, USA). The remaining chemicals and reagents used were of analytical grade.

### 2.2. Sample Collection and Extraction

Tender branches and leaves of *M. oleifera* were collected from Suncheon Bay Moringa Cooperative (Suncheon-si, Jeollanam-do, Korea), chopped into about one-centimeter pieces, air-dried under room temperature, and stored in air-tied polythene bags for further uses. A part of the air-dried material was pulverized into powder by using IKA MF10 laboratory pulverizer (Staufen, Germany). Based on the DPPH radical scavenging activity and flavonoid content, one of the previous studies revealed that the 100 °C water in a pressurized system was the optimum temperature for *M. oleifera* leaf extraction [23]. In this study, boiling water (900 mL) was used to extract 100 g of powder at 100 °C for 4 h. After centrifugation and filtration, the filtrate was frozen at −80 °C and freeze-dried to obtain a dry powder of hot water extract. The freeze-dried powder was kept in an air-tight container at −20 °C.

### 2.3. Compositional Analysis of MOH

The total polyphenolic content of MOH was determined using the method outlined by Singleton et al. (1999) [24]. A gradient concentration of gallic acid was used as the reference standard. The Lowry method with BSA as the reference standard was used to determine total protein content in MOH [25]. Carbohydrate content was measured according to the phenol–sulphuric method using d-glucose as the reference standard [26].

### 2.4. High-Performance Liquid Chromatography (HPLC) Analysis of MOH

HPLC analyses were conducted with the aid of a Shimadzu system equipped with a gradient pump integrated into an SPD-M30A Photodiode Array Detector. The separation was achieved by a Luna PFP (2) 100A (150 × 3.0 mm, 3 m) column. The system was eluted with the gradient program of a binary solvent system consisting of 0.1% formic acid in water (A) and 0.1% formic acid in methanol (B) mixture at a constant flow rate of 0.34 mL/min. The sample injection volume was 3 µL. A 5-min post-run at starting conditions was performed to equilibrate the column. The gradient program was started with 0% of B and then changed to obtain 25%, 45%, 65%, 85%, and 100% of eluent B at 0, 25, 50, 75, 100, and 125 min, respectively. For each predetermined phenolic compound that was evaluated, the mixed standard solution was prepared by diluting the mixed stock standard solutions in methanol to provide a concentration of 1.176 mg/mL. A solution of MOH at a concentration of 10 mg/mL in methanol was prepared, sent through a syringe filter, injected, and phenolic compounds were evaluated using UV absorbance at 270 nm. The phenolic compounds in the MOH were identified by comparing retention times and absorbance spectrum profiles with the standards of each detected compound. Quantification was carried out by comparing the chromatograms of the standard mixture with that of the sample (MOH). Triplicate independent analyses were conducted, and the results are presented as means ± standard deviations from three separate studies. All chromatographic processes were carried out at 35 °C, with data acquisition, peak integration, and calculations carried out using LCsolution version 1.24 SP2 software (Shimadzu, Kyoto, Japan).

### 2.5. Radical Absorbance Capacity of MOH

ABTS^+^ and DPPH radical scavenging activity of MOH were determined using the methods described in one of the previous studies conducted by Um et al. (2017) [27]. In brief, the stock solution containing ABTS, and potassium persulfate (K_2_S_2_O_8_) were mixed with each 50 μL of the MOH hydrolysates (250 μg/mL). Then the mixture was left to react for 10 min in the dark, and the absorbance was measured at 414 nm by using a SpectraMax M2 microplate reader (Molecular Devices, Sunnyvale, CA, USA). To analyze the DPPH radical scavenging capacity, 100 μL of MOH hydrolysates (250 μg/mL) was added to 100 μL of DPPH solution (150 μM). The mixture was kept in dark at room temperature for 30 min, and then absorbance was measured at 517 nm by using a SpectraMax M2 microplate reader. Ferric chloride (10% in distilled water) was used to measure the Ferric reducing antioxidant power (FRAP) of MOH. Briefly, 0.1 M phosphate buffer (pH 6.6–7.0), 1% potassium ferricyanide, and sample or distilled water as a control mix in a 3:5:2 ratio, vortexed and incubated for 20 min at 50 °C. Thereafter, 10% Trichloroacetic acid was mixed and centrifuged at 3000 rpm for 10 min. The supernatant was taken, mixed with distilled water and 10% ferric chloride in a 5:5:1 ratio, and the absorbance was measured at 700 nm by using SpectraMax M2 microplate reader. Oxygen radical absorbance capacity (ORAC) assay was used to examine the antioxidant ability of MOH. In brief, 50 μL of 50 g/mL MOH in 75 mM phosphate buffer (pH 7.0) was mixed with 78 nM fluorescein (440 g/mL in 75 mM phosphate buffer) and incubated at 37 °C for 15 min. Then, 25 μL of AAPH was added to each well, and the emission at 538 nm under 485 nm excitation was recorded every 5 min for 2 h using SpectraMax M2 microplate reader. Trolox (1, 5, 10, 20, and 40 μM in 75 mM phosphate buffer) was used as the standard. The antioxidant capacity of MOH was expressed as Trolox equivalents per μg/mL of MOH.

### 2.6. Cell Culture

Vero cells (The monkey kidney fibroblasts, KCLB, Seoul, Korea) were cultured and then sub-cultured every 3 days in a humidified atmosphere at 37 °C with 5% of CO_2_ in DMEM supplemented with 1% P/S and 10% inactivated FBS. Cells were seeded in 24-well plates or 96-well plates accordingly for subsequent experiments.

### 2.7. Cell Viability and ROS Production Analysis

Vero cells seeded in a 96-well plate were treated with a series of MOH concentrations and incubated for 1 h. Then, 10 μL of H_2_O_2_ (1 mM) was added and incubated at 37 °C for 24 h. Cells were then subjected to MTT assay. The absorbance of formazan crystals dissolved in DMSO was measured by using SpectraMax M2 microplate reader at 570 nm. Effect of MOH on intracellular ROS levels in H_2_O_2_-induced Vero cells was measured by 2′,7′-dichlorofluorescein diacetate (DCF-DA) assay. In brief, seeded cells were incubated for 24 h and then treated with a series of concentrations of MOH for one hour. Then, 10 μL of H_2_O_2_ (1 mM) was added to each well and incubated for another 1 h. Finally, these cells’ intracellular ROS levels were determined after adding 10 μL DCF-DA (500 μg/mL) by using a microplate reader, and images were captured by using Invitrogen™ EVOS™ M5000 fluorescence microscope (Thermo Fisher Scientific, Waltham, MA, USA).

### 2.8. Evaluation of Apoptotic Body Formation

The nuclear morphology of the H_2_O_2_-induced Vero cells was observed to identify the apoptotic body formation. For that, seeded cells were treated with a series of MOH concentrations, incubated for 1 h, and then treated with 1 mM H_2_O_2_. After 24 h, cells were stained with 10 μL of Hoechst 33,342 (0.5 mg/mL) and propidium iodide (2.5 μM). After 10 min of incubation, nuclear morphology was examined by using Invitrogen™ EVOS™ M5000 fluorescence microscope.

### 2.9. Cell Cycle Analysis

The cell cycle was investigated according to the procedure described in one of the previous studies [28]. In brief, the cells were rinsed with PBS and permeabilized in 70% ethanol for 30 min after harvesting. Then, the cell pellets were centrifuged again after being gently resuspended in PBS containing EDTA. For 30 min, the fresh pellet was gently resuspended in PBS solution containing PI, EDTA, and RNase A. The cells were then examined using a Beckman Colter CytoFLEX system flow cytometer (Brea, CA, USA).

### 2.10. Mitochondrial Depolarization Analysis by JC-1 Assay

Cultured cells were harvested after 4 h of sample treatment followed by stimulation and the mitochondria membrane potential was measured using MitoProbe JC-1 Assay Kit (Thermo Fisher Scientific, Waltham, MA, USA) according to the manufacturer’s instructions followed by flow cytometric analysis.

### 2.11. Western Blot Analysis

Cells were seeded in 10 cm culture dishes for 24 h at 2 × 10^5^ cells/mL concentration and stimulated with 1 mM H_2_O_2_ after being treated with 15.6, 31.3, and 62.5 μg/mL of MOH for 2 h. Herein, 50 μM vitamin C was used as a positive control. Then, the cells were harvested for western blot analysis. Insoluble materials were removed from cell lysate by centrifugation followed by lysis. Cells were lysed by a nuclear and cytoplasmic extraction kit, NE-PER^®^ (Thermo Scientific, Rockford, IL, USA). The protein concentrations in cell lysate were estimated using a BCA protein assay kit (Thermo Scientific, Rockford, IL, USA). After estimation was completed, 30 μg of protein of each lysate were subjected to electrophoresis on 10% polyacrylamide gels. Resolved protein bands were transferred onto nitrocellulose membranes (Merck Millipore, Dublin, Ireland), blocked with 5% skim milk in TBST, and then incubated with primary antibodies and HRP-conjugated secondary antibodies. Then, identified protein bands were visualized by an enhanced chemiluminescence (ECL) western blotting detection kit followed by imaging on the Core Bio Davinch-Chemi^TM^ imaging system (Seoul, Korea).

### 2.12. Statistical Analysis

All the relevant data were presented as the mean ± standard error of the mean (SEM), while all statistical analyses were performed using the SPSS software (Version 24.0, Chicago, IL, USA). The values were evaluated and significant variations among data sets were obtained by using one-way analysis of variance (ANOVA) followed by Duncan’s multiple range tests, and *p* < 0.05 was considered as statistically significant.

## 3. Results

### 3.1. Extraction Yield and Proximate Composition of MOH

According to the results indicated in Table 1, the polysaccharide content was relatively higher than the polyphenol composition and protein content of MOH on a dry basis (%).

### 3.2. Composition of Antioxidant Phytochemicals in MOH

HPLC was used to examine phenolic compounds in MOH. The chromatograms obtained by the MOH were compared to seventeen distinct phenolic standards that had been predetermined (Figure S1). HPLC analysis revealed the presence of fourteen out of seventeen phenolic standards in the MOH. D-mandelic acid, gentisic acid sodium salt hydrate, and syringic acid were not detected in the MOH, but 3-hydroxy benzoic acid was identified in the greatest concentration of 95.64 ± 0.36 μmol/100 g, and rutin hydrate was found in the lowest concentration, which was 1.11 ± 0.43 μmol/100 g, as shown in Table 2. Corresponding chromatograms are provided with Supplementary Materials (Figure S1).

### 3.3. Antioxidant Activities of MOH

**Figure 1:**
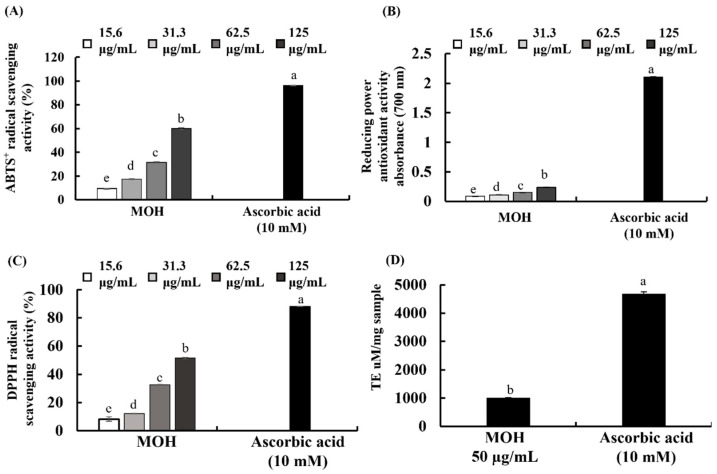
Antioxidant activities of MOH. (**A**) ABTS^+^ radical scavenging activity, (**B**) FRAP of MOH, (**C**) DPPH radical scavenging activity, and (**D**) ORAC of MOH. Ascorbic acid (Vit C, 10 mM) was used as the positive control. All experiments were performed in triplicate (*n* = 3) to determine if repeatability and lettered error bars were significantly different (*p* < 0.05).

The decolorization of the ABTS^+^ extent is determined by the percentage inhibition of the ABTS^+^ radical cations [29,30]. DPPH absorbs at 515 nm in the radical form, but this absorption is reduced when it is reduced by an antioxidant or a radical species [29,30]. In the FRAP, the antioxidant activity of an extract is determined based on the ability to reduce ferric (III) iron to ferrous (II) iron. MOH indicated substantial ABTS^+^ and DPPH radical scavenging activity with respective IC_50_ values of 102.52 and 122.55 µg/mL (Figure 1A,C). Scavenging activity increased with the MOH concentration, with a maximum scavenging activity of 60.05 ± 0.24% and 51.48 ± 0.41%, respectively, at 125 μg/mL. The MOH had a significantly low FRAP value compared to the positive control, vitamin C (Figure 1B). The FRAP of the MOH indicated a significant and dose-dependent increase with a maximum absorbance of 0.2398 at 125 μg/mL (Figure 1B). The ORAC of the MOH was 1004.95 µmol TE/mg of the sample, which is illustrated in Figure 1D.

### 3.4. Effects of MOH on Cell Viability and Intracellular ROS Production

**Figure 2:**
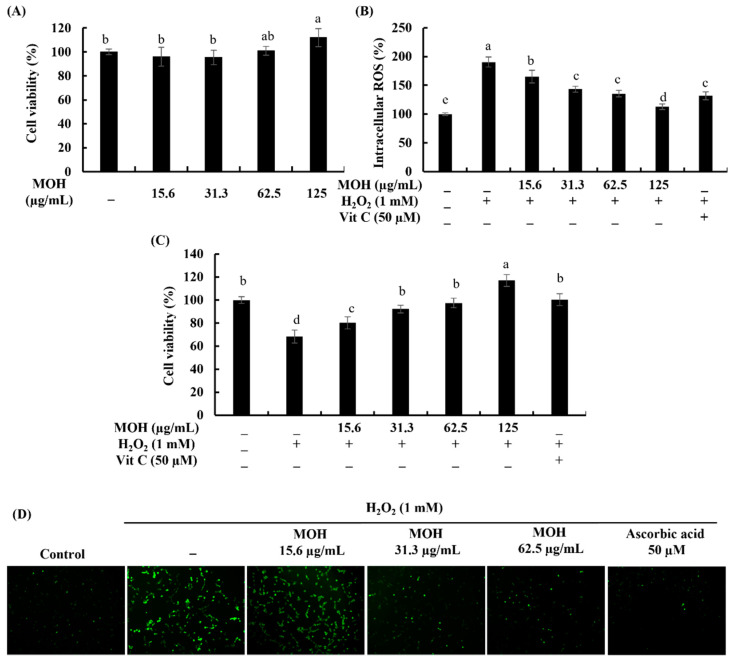
Cytoprotective effects of MOH against H_2_O_2_-induced Vero cells. (**A**) Cytotoxicity, (**B**) intracellular ROS generation, (**C**) cell viability, and analysis of ROS generation through (**D**) fluorescence microscopy with 2′,7′-dichlorofluorescein diacetate (DCFH-DA) staining of MOH-pretreated H_2_O_2_-induced Vero cells. Ascorbic acid (Vit C, 50 μM) was used as the positive control. All experiments were performed in triplicate (*n* = 3) to determine if repeatability and lettered error bars were significantly different (*p* < 0.05).

According to measured intracellular ROS inhibition in Vero cells, the MOH indicated good antioxidant effects. As shown in Figure 2A, the MOH concentrations used were not cytotoxic towards the Vero cells up to 125 μg/mL. Therefore, the concentrations of 15.6 μg/mL, 31.3 μg/mL, and 62.5 μg/mL were used throughout the study. Contrary to this, the cell viability of H_2_O_2_-induced Vero cells was decreased compared to control cells, while MOH-treated cells showed an increase in dose-dependent cell viability (31.3–125 μg/mL) (Figure 2C). Vitamin C was used as a positive control. H_2_O_2_ boosted the intracellular ROS production in Vero cells and showed a reduction in ROS production in a dose-dependent manner by MOH-pretreated cells (Figure 2B). Figure 2D indicates the fluorescence microscopy analysis of the inhibitory effects of the MOH against H_2_O_2_-induced oxidative stress in Vero cells. According to the results, H_2_O_2_-stimulated cells unveiled higher green fluorescence for DCFH-DA compared to the control group. MOH-pretreated Vero cells indicated a dose-dependent reduction in the green fluorescence supporting the potential antioxidant activities of MOH against H_2_O_2_-induced intracellular ROS generation in Vero cells.

### 3.5. Effect of MOH on H_2_O_2_-Induced Apoptosis

**Figure 3:**
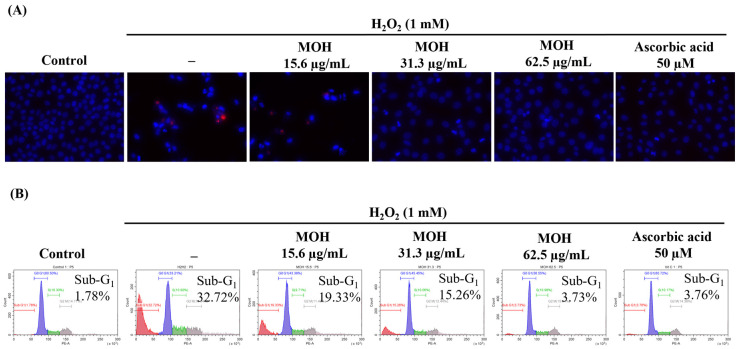
Effect of MOH on H_2_O_2_-induced apoptosis in Vero cell. (**A**) Evaluation of apoptotic body formation and necrosis by Hoechst 33342 and PI Nuclear double staining. (**B**) Analysis of Sub-G_1_ apoptotic populations by flow cytometry using PI. All experiments were performed in triplicate (*n* = 3) to determine repeatability.

In the Hoechst (33342) and PI nuclear double staining, cells with equally stained nuclei are considered viable cells, where chromatin condensation and fragmentation indicate apoptosis, and red to orange nuclei indicate necrotic death of cells [31]. As Figure 3A indicates, H_2_O_2_-induced Vero cells indicated nuclear condensation and fragmentation. Dose-dependent treatment of the MOH reduced the formation of the apoptotic bodies by reducing the condensation of chromatin and nuclear fragmentation. H_2_O_2_-induced Vero cells without pretreatment of the MOH showed necrotic cells. These results were compatible with the results of the cell cycle analysis conducted with flow cytometry. Cell cycle analysis was performed after PI staining to test if the cells were undergoing apoptosis. The hypodiploid cell population in the Sub-G_1_ phase was analyzed by flow cytometry. According to the findings (Figure 3B), an increase in the Sub-G_1_ apoptotic cell population (32.72%) further indicates H_2_O_2_-induced apoptosis compared to the non-stimulated control (1.78%). The MOH treatment dose-dependently reduced the Sub-G_1_ apoptotic cell population.

### 3.6. Effect of MOH on Mitochondrial Depolarization and Expression Levels of the Proteins Related to Mitochondrial Apoptosis Pathway

**Figure 4:**
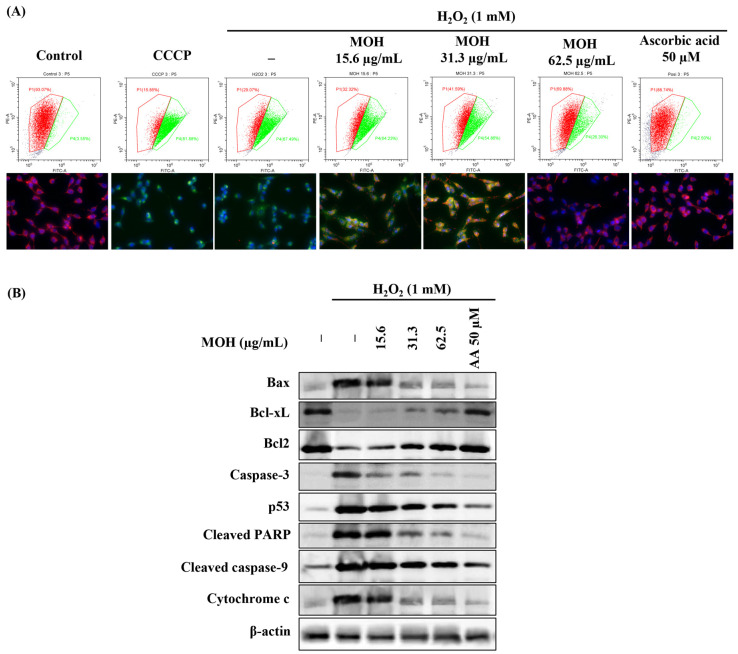
Effect of MOH on (**A**) mitochondrial depolarization and (**B**) variation in mitochondria-mediated apoptotic pathway proteins expression levels against H_2_O_2_-induced apoptosis in Vero cell. All experiments were performed in triplicate (*n* = 3) to determine repeatability.

The JC-1 (5,5′,6,6′-tetrachloro-1,1′,3,3′-tetraethylbenzimidazolyl carbocyanine iodide) has been used for many years as a specific membrane-permeable dye for measuring mitochondrial membrane potential. Emission of the red color indicates healthy mitochondria. The decreased potential of the mitochondrial membrane causes a significant shift in the blue laser excitation from orange to green fluorescence emissions at 488 nm. Based on the data shown in Figure 4A, a comparatively large number of cells (93.07%) indicated red fluorescence in the control. Stimulated cells denoting the lowest number of cells with red fluorescence represent that H_2_O_2_ increases the population of hyperpolarized mitochondria in H_2_O_2_-induced Vero cells. Moreover, Figure 4A exhibits fluorescence microscopic images that indicate the visual condition of the cells used in flow cytometry. MOH treatment dose-dependently decreased the population of hyperpolarized mitochondria in H_2_O_2_-induced Vero cells, whereas 62.5 μg/mL of MOH showed the best protective effect against H_2_O_2_. The positive control, vitamin C indicated the highest protective effect against H_2_O_2_ in Vero cells at 50 μM.

Results from nuclear double staining with Hoechst and PI and JC-1 assay, predicted that apoptosis in H_2_O_2_-induced Vero cells happened through a mitochondria-mediated apoptotic pathway. To examine the mitochondria-mediated apoptosis, levels of Bcl-xL, Bcl-2, Bax, caspase 3, p53, cleaved PARP, cleaved caspase 9, and cytochrome c were investigated by using western blotting. Figure 4B shows that Bcl-xL and Bcl-2 were suppressed and Bax, caspase 3, p53, cleaved PARP, cleaved caspase 9, and cytochrome c were increased by H_2_O_2_ in Vero cells. Hence, MOH controls the mitochondria-mediated apoptosis by increasing the levels of Bcl-xL and Bcl-2 and decreasing the levels of BAX, Caspase 3, p53, cleaved PARP, cleaved caspase 9, and cytochrome c (Figure 4B). The suppression of apoptosis was mediated via the mitochondrial apoptosis pathway.

### 3.7. Effect of MOH on Activation of Nrf2/HO-1/NQO1 Signaling Pathway

**Figure 5:**
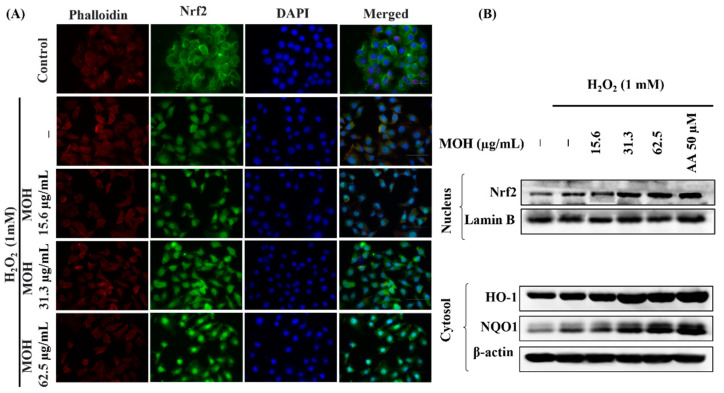
(**A**) Immunofluorescence analysis of Nrf2 nuclear translocation and (**B**) dose-dependent variation of the effects of MOH on Nrf2-mediated activation of HO-1 and NQO1 in H_2_O_2_-induced Vero cells.

Activation of the nuclear factor erythroid 2-related factor 2 (Nrf2)/heme oxygenase 1 (HO-1) signaling pathway has been shown to reduce ROS generation in cells in previous research [32]. The presence of green fluorescence in the nucleus can be used to identify Nrf2 nuclear translocation in an immunostaining experiment. The strong green fluorescent signal implies that Nrf2 nuclear translocation is increasing. Figure 5A shows that pretreatment with the MOH enhanced Nrf2 signaling in H_2_O_2_-stimulated Vero cells by boosting Nrf2 nuclear translocation in a dose-dependent manner. According to the results of western blot analysis, H_2_O_2_-stimulation raised the levels of nuclear Nrf2 as well as cytosolic HO-1 and NQO1 in Vero cells, followed by a substantial and dose-dependent increase in the levels of nuclear Nrf2 (Figure 5B).

## 4. Discussion

The existence of selected phenolic components in *Moringa oleifera* hot water extract (MOH) and its antioxidant activity against H_2_O_2_-induced oxidative stress in Vero cells were investigated in this study. The hot water extraction method was selected to extract *M. oleifera* because of its efficiency with a reduced cost. In addition, the hot water extraction method is useful in industry and can be used to extract ingredients for food and cosmetics [33]. Based on the outcomes of the study, the extraction yield of MOH was 35.67 ± 0.44%, while the previous study reported a yield of 28.26% per 90 min soaking at 121 ± 0.5 °C water [33]. According to the current observations, MOH had a relatively high polysaccharide content compared with protein while having a comparatively low polyphenol content. The present study reports polyphenol content of 5.24 ± 0.07% (*w*/*w*) in MOH, while Sreelatha, S. and Padma, P.R. (2009) reported 4.58% in Soxhlet extraction for 18–20 h of *M. oleifera* leaves [34]. Polyphenols are recognized as antioxidants due to their hydroxyl groups’ capacity to scavenge free radicals, which have a significant in vitro effect [34,35]. Glucosinolates, and potentially alkaloids, in addition to polyphenols, are thought to be responsible for the bioactive effects of *M. oleifera* as reported before [36]. Pawlowska, E. et al. particularly mentioned the presence of two hydroxyl groups in their B ring structure in some dietary polyphenols, such as quercetin and cyanidin-3-glucoside affect directly to scavenge ROS in cells [37]. Young Chool Boo reported that the phenolic compounds derived from a variety of plants can lower the levels of ROS in cells and/or enhance cellular antioxidant capacity and anti-inflammatory effects [38]. Aside from direct ROS scavenging, dietary phenols can reduce oxidative stress through a variety of mechanisms such as inducing Nrf2 activation [37]. Kanner J.’s review contains evidence that polyphenols can inhibit cellular protein tyrosine phosphatases and activate cell signaling through transcription of the Nrf2 axis to adapt and protect cells against oxidative stress by producing H_2_O_2_ at low levels (1–10μM) [39]. In this study, the presence of gallic acid, 2,3,4-trihydroxy benzoic acid, 3,4-dehydroxy benzaldehyde, 4-hydroxy benzoic acid, catechin hydrate, vanillic acid, 3-hydroxy benzoic acid, chlorogenic acid, p-coumaric acid, 3,4 dimethoxy benzoic acid, sinapic acid, rutin hydrate, trans-cinnamic acid, and quercetin in the MOH were confirmed based on the results obtained from HPLC analysis (Supplementary Material Figure S1). According to the results, the total polyphenolic content on a weight basis was found to be 136.28 mg/100 g. Quercetin-like phenolics were present in the MOH, which can be implicated to have positive benefits on human health and to alleviate oxidative stress [40]. Based on the findings, a quantitative difference was observed between total polyphenolic content as determined by proximate composition analysis and HPLC analysis. This suggests that MOH may contain a range of additional polyphenolic compounds, which was not revealed by HPLC analysis in this study. Due to the presence of phenolic compounds in MOH, H_2_O_2_ production in Vero cell-cultured media after treatment of MOH for 1 h was analyzed. According to the results obtained from the analysis, it revealed that MOH produces a maximum quantity of 0.235 ± 0.033 μM of H_2_O_2_ in cell culture media at 62.5 μg/mL of MOH concentration (Supplementary Material Figure S3). In this study, TLC analysis (Supplementary Material Figure S2) verified the existence of antioxidative chemicals in MOH seen from the change of color in KMnO_4_-stained TLC. The discoloration of KMnO_4_ is caused by a reduction in the oxidative state of Mn from +7 to +2 [41]. Furthermore, the occurrence of nucleophiles, aldehydes, and ketones was confirmed by *p*-anisaldehyde and Vanillin stains, which commonly undergo Aldol condensation and acetalization [42]. UV light with a short wavelength and long wavelength (254 nm and 365 nm, respectively) to enable visualization of TLCs, can be used to identify extended conjugated (aromatic) systems [42]. Bright purple or blue fluorescent represents highly-conjugated compounds, which may be comprised of a single or multiple aromatic rings. Accordingly, the presence of single or multiple aromatic rings in the MOH was confirmed by UV-enabled visualization of TLCs. Unsaturated and aromatic compounds were visualized in a brown color by iodine stains. Saponins and phenols were visualized by the 10% sulphuric acid in ethanol and the ferric chloride stains, respectively [42].

The ABTS^+^ diammonium salt radical cation decolorization test is one of the commonly used spectrophotometric methods to evaluate the antioxidant activity of numerous substances, including plant extracts [43]. 2′,7′-dichlorodihydrofluorescein (DCFH) is highly sensitive to ROS and can be oxidized to a highly fluorescent 2′,7′-dichlorofluorescein (DCF) by ROS [44]. DCF can be seen with green fluorescence in fluorescence microscopic observations. During reactions between DPPH radicals and antioxidative extracts or antioxidative compounds, the absorption reduction with a characteristic wavelength is widely used to evaluate antioxidative activity that can give hydrogen [45]. The ABTS assay revealed that ABTS+ radical scavenging activity increases dose-dependently with the MOH. Antioxidant capabilities in fruit, vegetables, and other plant materials as well as their products, have been determined using the DPPH and ORAC assays. Because it uses a biologically relevant radical source, the ORAC assay is believed to be more relevant [46]. The finding of the ABTS assay was strengthened by DPPH scavenging activity and an ORAC value of the MOH. Further analyses were conducted to evaluate the protective effect of the MOH against H_2_O_2_-induced oxidative stress in Vero cells based on the promising radical scavenging capacity exhibited by the MOH.

H_2_O_2_ treatment raised intracellular ROS levels in cells, resulting in enhanced oxidative stress in Vero cells. The results of the present MTT and DCF-DA analysis indicate that MOH has promising antioxidant and protective effects in Vero cells against the H_2_O_2_-induced oxidative stress. The outcomes of the investigation of DCF-DA-stained cells by fluorescence microscopy indicated parallel results to the fluorometric analysis of Sub-G_1_ apoptotic populations, revealing the effects of the MOH against H_2_O_2_-induced oxidative stress. Based on simultaneous DCF fluorescence changes, one of the previous studies confirmed flavonoids’ cell membrane permeability and disclosed that polyphenolic compounds (particularly myricetin in the relevant study) can diffuse through the cell membrane into the cells, where they prevent the production of different ROS compounds in the polar intracellular environment [47]. In that sense, the antioxidant effect of the MOH on cells can be justified since it contains numerous phenolic compounds.

Chromatin condensation, membrane surface hemorrhage, phosphatidylserine excretion, DNA fragmentation, and eventual apoptotic body formation are specific morphological and biochemical features associated with apoptosis [48]. Results obtained from the nuclear double staining analysis with Hoechst and PI in this study indicated the effects of MOH on the reduction of the events of apoptosis, including the formation of the apoptotic bodies and DNA damage. The above observations can be presented as evidence of the protective potential of MOH on Vero cells against oxidative stress induced by H_2_O_2_. Moreover, the above finding was confirmed by the reduction in Sub-G_1_ apoptotic cells’ accumulation along with the results of the JC-1 assay. Collectively, the above evidence suggested the effectiveness of MOH in ameliorating apoptosis in H_2_O_2_-induced Vero cells and the connection between the inhibition of mitochondrial dysfunction and H_2_O_2_-induced apoptosis progression. Based on the above outcomes, further experiments were designed to obtain a better understanding of the role of MOH in apoptosis pathway regulation.

The mitochondria-mediated caspase activation apoptosis pathway is one of the major signaling pathways that mediate apoptosis in the mammalian cells, with the characteristic permeabilization of the mitochondrial external membrane and consequent release of cytochrome c to the cytoplasm [49]. Many studies have reported that the regulation of this pathway depends on various factors including the type of apoptotic stimulant as well as the type of cell undergoing apoptosis [50]. Suppression of the above pathway reduces apoptosis, thereby minimizing the relevant responses such as limiting non-communicable illnesses. Bcl-2 family proteins including antiapoptotic Bcl-2 and Bcl-xL, and pro-apoptotic proteins including Bax, Bak, Bok, Bad, Bid, Bik, Bim, Bmf, Puma, and Noxa are identified as major responsible proteins that control mitochondrial outer membrane permeabilization [50]. The activation of caspase 9 causes the progression of apoptosis. Caspase 9 becomes cleaved caspase 9 when it meets cytochrome c. Mitochondrial pathway-mediated apoptosis is further aggravated by pro-apoptotic stimuli inducing p53, activated caspase-9 and, consequently, activating downstream effector caspases such as caspase −3, −6, and −7. The catalytic activity of PARP is inhibited by effector caspases by cleaving PARP. The present study clarified the effect of H_2_O_2_ on the initiation of mitochondria-mediated apoptosis proteins and the dose-dependent attenuation effects of MOH.

The Nrf2–Keap1 system is a physiological thiol-based sensor-effect system in eukaryotes that responds to oxidative stress by maintaining redox homeostasis [5]. Nrf2 has been identified as the primary regulator of the transcription of several antioxidant and cytoprotective genes, whereas regulating oxidative stress and securing physiological homeostasis requires the regulation of antioxidant gene expression. Nrf2 is typically found in the cytosol due to its interaction with a cytosolic actin-binding protein called Keap1 (Kelch-like ECH-associated protein 1), also known as INrf2 (inhibitor of Nrf2) [51]. When cells encounter stress, such as exposure to mild oxidants, Nrf2 dissociates from Keap1, becomes stabilized, and translocases into the nuclei, and promotes the antioxidant response element-driven antioxidant gene transcription of numerous antioxidant genes, including NQO1, HO-1, glutathione S-transferase A2, and glutamate-cysteine ligase [52]. The results of the immunofluorescence analysis of Nrf2 nuclear translocation in H_2_O_2_-induced Vero cells reveal that Nrf2 activation and nuclear translocation were increased by H_2_O_2_ stimulation and dose-dependently upregulated by the MOH in Vero cells. Apart from the scavenging of ROS in the cytosol by phenolic compounds, several studies have revealed that the nanomolar range of H_2_O_2_ generated in the cell growth media could be penetrating cells, activating Nrf2 signaling, and supporting to adapt to changes in the environment and oxidative stress [39,53]. Moreover, H_2_O_2_ is acknowledged as the most important ROS in the redox regulation of biological processes [5]. The activation of Nrf2 signaling in Vero cells can be explained by the fact that MOH generated low levels of H_2_O_2_ in the cell growth medium after MOH incubation. Furthermore, western blot analysis, which was conducted to investigate the effect of MOH on H_2_O_2_-induced Vero cells, shows a significant increment of expression levels of nuclear Nrf2, cytosolic HO-1, and NQO1 with the MOH strengthened the antioxidant activity of the MOH on H_2_O_2_-induced Vero cells.

## 5. Conclusions

Based on the present study, hot water extract of *M. oleifera* (MOH) has various phenolic compounds that possess potent antioxidant activity and have an ability to generate a low level of H_2_O_2_ in Vero cell-cultured media, which collectively ameliorate H_2_O_2_-induced oxidative stress and apoptosis in Vero cells by suppressing the mitochondria-mediated apoptosis pathway and regulating the Nrf2/HO-1 signaling pathway. Further analysis of MOH bioactivity and safety would potentiate its applications in the manufacturing of functional foods with beneficial bioactivities centered on their antioxidant activity.

## Figures and Tables

**Table 1:** Composition of MOH.

MOH	Composition (%)
Yield	35.67 ± 0.44
Protein	19.01 ± 0.27
Polysaccharide	44.95 ± 0.66
Polyphenol	5.24 ± 0.07

Mean ± SEM (all experiments were performed in triplicate (*n* = 3) to determine the repeatability).

**Table 2:** Availability of the examined phenolic compounds in MOH.

Phenolic Compound	μmol/100 g
Gallic acid	78.24 ± 0.18
D-mandelic acid	Not detected
2,3,4-trihydroxybenzoic acid	79.00 ± 0.06
3,4-dehydroxybenzaldehyde	96.87 ± 0.43
4-hydroxybenzoic acid	97.31 ± 0.14
Gentisic acid sodium salt hydrate	Not detected
Catechin hydrate	43.34 ± 0.26
Vanillic acid	79.52 ± 0.36
3-hydroxy benzoic acid	95.64 ± 0.36
Chlorogenic acid	35.73 ± 0.03
Syringic acid	Not detected
p-coumaric acid	81.07 ± 0.12
3,4 dimethoxy benzoic acid	18.33 ± 1.26
Sinapic acid	60.08 ± 0.13
Rutin hydrate	1.11 ± 0.43
Trans-cinnamic acid	91.66 ± 0.00
Quercetin	45.89 ± 0.07

Mean ± SEM (all experiments were performed in triplicate (*n* = 3) to determine the repeatability).

## Data Availability

The data presented in this study are available on request from the corresponding author.

## Supplementary Materials

The following supporting information can be downloaded at: https://www.mdpi.com/article/10.3390/foods11030420/s1, Figure S1: HPLC chromatography of MOH, Figure S2: Thin-layer chromatography (TLC) analysis of MOH, Figure S3: H_2_O_2_ production levels in Vero cell-cultured media after treatment of MOH for one hour.
