# Supplementary material for: Moringa oleifera Hot Water Extract Protects Vero Cells from Hydrogen Peroxide-Induced Oxidative Stress by Regulating Mitochondria-Mediated Apoptotic Pathway and Nrf2/HO-1 Signaling
Source: Foods. 2022 Jan 31;11(3):420. doi: 10.3390/foods11030420 (PMC8834631; doi:10.3390/foods11030420)
Supplement: Supplementary file 1 [file foods-11-00420-s001.zip › foods-1535070-supplementary.pdf]

Supplementary material

Moringa oleifera hot water extract protects Vero cells from hydrogen peroxide-induced oxidative stress by regulating mitochondria-mediated apoptotic pathway and Nrf2/HO-1 signaling

S1.HPLC chromatography

A.

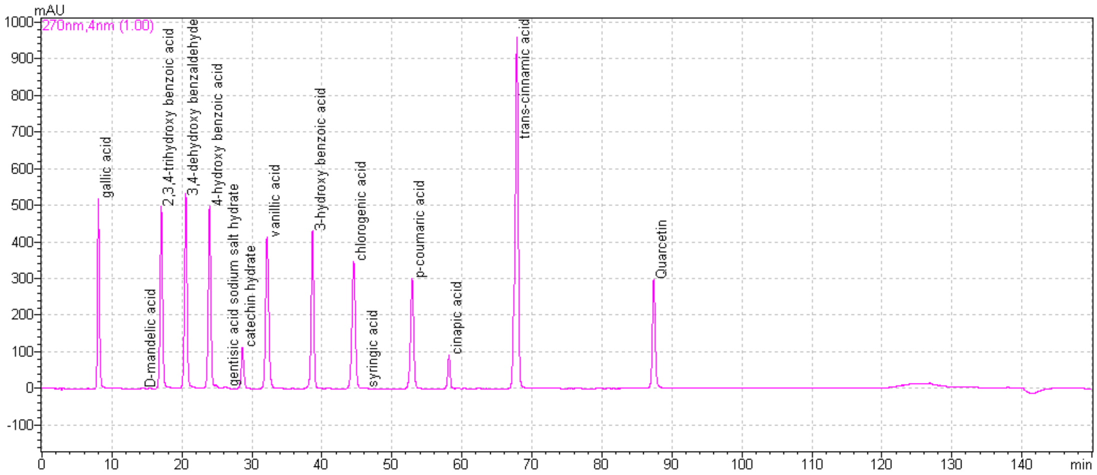

B.

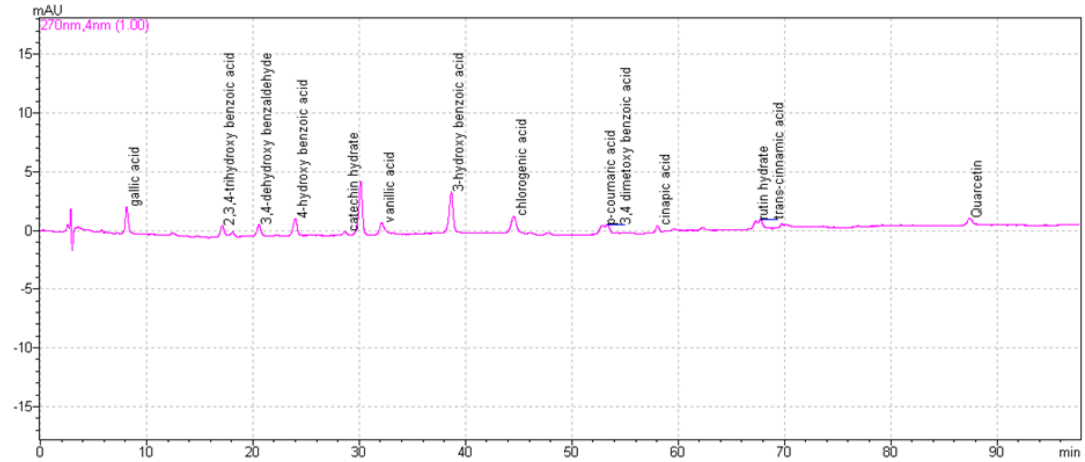

Figure S1. Chromatograms of gallic acid, D-mandelic acid, 2,3,4-trihydroxy benzoic acid, 3,4-dehydroxy benzaldehyde, 4-hydroxy benzoic acid, gentisic acid sodium salt hydrate, catechin hydrate, vanillic acid, 3-hydroxy benzoic acid, chlorogenic acid, syringic acid, p-coumaric acid, 3,4 dimetoxy benzoic acid, cinapic acid, rutin hydrate, trans-cinnamic acid, and quarcetin obtained from HPLC analysis of A. Standard phenolic compound mixture and **B.** MOH.

S2.Thin-layer chromatography (TLC) analysis of MOH

TLCs (reversed-phase/ C18) were developed using 70% methanol in distilled water as the mobile phase solvent system after spotting MOH. Preliminary studies were conducted to select ideal solvent systems for the TLC analysis. TLCs were visualized under UV illumination using a CN-6 ultraviolet lamp (Cedex, France) at 365 and 254 nm wavelengths and by numerous staining methods; 10% sulphuric acid, iron (III)chloride, iodine, p-anesaldehyde, potassium permanganate, vanillin sulfate with or without heating.

TLC analysis

Active constituents in the MOH were analyzed by TLC. According to the results (Figure 1.), the MOH may contain compounds that can be correlated with antioxidants activity detected by Potassium permanganate ($KMnO_4$). The occurrence of one or more alcohols, amines, aldehydes, and ketone functional groups in MOH was confirmed by visualizing p-Anisaldehyde and vanillin stains. Furthermore, the existence of unsaturated and aromatic compounds, saponins, and phenols in MOH was definite by the above results.

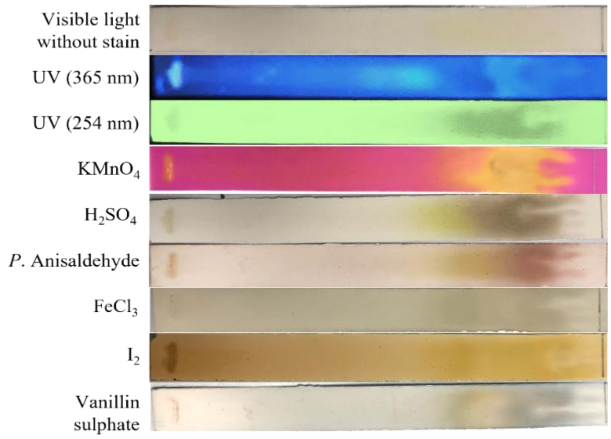

Figure S2. TLC analysis of MOH in methanol: water (7:1) solvent system. Ultraviolet (UV) illumination at 365 and 254 nm wavelength, staining systems including $KMnO_4$,10% H_2SO_4 in methanol, p-anisaldehyde, $FeCl_3$, I_2, and vanillin sulfate were used to the visualization of the strips.

S3. H_2O_2 production in MOH treated cell culture media

H_2O_2 production in Vero cell-cultured media after MOH treatment was investigated by using EZ-Hydrogen peroxide assay kit (DoGenBio Co.Ltd, South Korea). In brief, Vero cells were seeded in a 24-well plate with DMEM media. After 24 hrs of incubation in a humidified atmosphere at 37°C with 5% of CO_2 in DMEM containing 1% P/S and 10% inactivated FBS, treated with the series of MOH concentrations and incubated for 1 hr. Then the formation of H_2O_2 in cell culture was measured by following the instructions given with the above-mentioned assay kit.

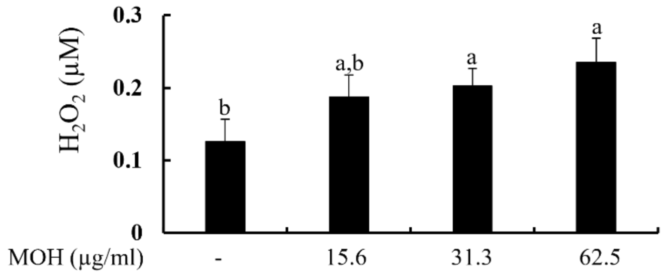

Figure S3. H_2O_2 production levels in Vero cell-cultured media after treatment of MOH for one hour. The experiment was performed in triplicate (n = 3) to determine if repeatability and lettered error bars were significantly different. (P<0.05).
